# Long term storage in liquid nitrogen leads to only minor phenotypic and gene expression changes in the mammary carcinoma model cell line BT474

**DOI:** 10.18632/oncotarget.16623

**Published:** 2017-03-28

**Authors:** Judit Fazekas, Thomas W. Grunt, Erika Jensen-Jarolim, Josef Singer

**Affiliations:** ^1^ Comparative Medicine, The Interuniversity Messerli Research Institute of the University of Veterinary Medicine Vienna, Medical University of Vienna and University of Vienna, Vienna, Austria; ^2^ Institute of Pathophysiology and Allergy Research, Center of Physiology, Pathophysiology and Immunology, Medical University of Vienna, Vienna, Austria; ^3^ Comprehensive Cancer Center, Department of Medicine I & Ludwig Boltzmann Cluster Oncology, Medical University of Vienna, Vienna, Austria; ^4^ Department of Internal Medicine II, University Hospital Krems, Karl Landsteiner University of Health Sciences, Krems, Austria

**Keywords:** long term storage in liquid nitrogen, cell line stability, BT474 cells, gene expression, EGFR

## Abstract

**Background/Aim:**

Cancer cell lines are indispensible surrogate models in cancer research, as they can be used off-the-shelf, expanded to the desired extent, easily modified and exchanged between research groups for affirmation, reproduction or follow-up experiments.

As malignant cells are prone to genomic instability, phenotypical changes may occur after certain passages in culture. Thus, cell lines have to be regularly authenticated to ensure data quality. In between experiments these cell lines are often stored in liquid nitrogen for extended time periods.

Although freezing of cells is a necessary evil, little research is performed on how long-term storage affects cancer cell lines. Therefore, this study investigated the effects of a 28-year long liquid nitrogen storage period on BT474 cells with regard to phenotypical changes, differences in cell-surface receptor expression as well as cytokine and gene expressional variations.

**Methods:**

Two batches of BT474 cells, one frozen in 1986, the other directly purchased from ATCC were investigated by light microscopy, cell growth analysis, flow cytometry and cytokine as well as whole-transcriptome expression profiling.

**Results:**

The cell lines were morphologically indifferent and showed similar growth rates and similar cell-surface receptor expression. Transcriptome analysis revealed significant differences in only 26 of 40,716 investigated RefSeq transcripts with 4 of them being up-regulated and 22 down-regulated.

**Conclusion:**

This study demonstrates that even after very long periods of storage in liquid nitrogen, cancer cell lines display only minimal changes in their gene expression profiles. However, also such minor changes should be carefully assessed before continuation of experiments, especially if phenotypic alterations can be additionally observed.

## INTRODUCTION

Cancer cell lines represent important surrogate models in cancer research [1] as primary malignant tissues are often very hard to access. Moreover, primary cancer tissue features high patient-to-patient heterogeneity, is hard to store and can often be acquired in small sample sizes only.

Cancer cell lines, however, can be used off-the-shelf, expanded to the desired quantity, easily transfected with genes of interest and can be widely distributed between laboratories and institutions in order to allow others to reproduce or continue experiments.

This routine includes numerous cycles of freezing and thawing. Considering that malignant cells need many mutations to gain the ability to grow outside the body as well as to gain immortality in this condition, these cell lines have very instable genomes [2]. Thus, these cells are very vulnerable for new mutations [3] with high potential to lose key features of the originally described cell line [4].

Even more disturbing are repeated reports of interchanged or cross-contaminated cell lines [5–6], which in some cases can be traced back for decades [7], annihilating research results of several years.

The International Cell Line Authentication Committee maintains a database on misidentified cell lines, which can be found online at www.iclac.org.

Therefore, international research communities have implemented guidelines for publication of cell line experiments [8]. Although these guidelines differ to some extent between scientific journals, all of them require cell lines to be authenticated in regular intervals. Cell line authentication methods comprise of short tandem repeat profiling (STR, [9]) as well as single nucleotide polymorphism arrays (SNP, [10–11]). Both methods are highly reliable, except in the case of mismatch repair deficient cell lines, which display microsatellite instability and therefore allelic drifts could be forced; if additionally selective outgrowth of otherwise persisting side lines occurs, STR-profiling can be misleading [11].

Nevertheless, in general, both authentication methods have helped to drastically reduce misleading results due to cross-contamination, or the fact that the surrogate model based on a respective cancer cell line is not valid any more, as the cell line has changed its phenotype substantially.

Another important point, however, which is not that thoroughly investigated yet, is the fact, that between experiments cell lines are stored in liquid nitrogen for very long periods of time. These storage conditions are completely unphysiological for cells. In order to reduce freezing damage, the addition of dimethyl sulphoxide (DMSO) to the freezing medium was already recommended by Lovelock & Bishop back in 1959 [12]. Since then, numerous efforts have been exerted to improve the viability of cells after the thawing process (reviewed in [13]).

Although it is unlikely that during storage mutations occur, it is not yet closely investigated to which extent changes in gene expression can occur. Only sporadic papers describe functional changes after cryopreservation, e.g. changes in IL-6 secretion of an adenocarcinoma cell line [14] or upregulation of caspase-3 in fibroblasts [15].

Thus, this study aims to investigate possible phenotypic changes, differences in cell surface receptor expression as well as cytokine and gene expression-variations of a standard breast cancer cell line, BT474 [16], after long-term storage in liquid nitrogen. We investigated two batches of BT474 cells, one directly purchased from the American Type Culture Collection (ATCC) and the other stored for 28 years in a research laboratory's liquid nitrogen tank.

## RESULTS

### Cell line authentication

Cell lines were sent to cell line authentication prior to the experiments. Firstly, short nucleotide polymorphism (SNP) - profiling was performed. Here, 24 gene loci were analysed; only if at least 22 loci can be amplified, DNA quality is rated “ok”, which was the case for both tested samples. As can be seen in Table 1, the SNP screening confirmed the identity of both cell lines with identical genotype codes and detected no cross-contamination.

**Table 1:** Cell line authentication reports of SNP profiling

*Sample Name*	*Cell line name*	*DNA quality*	*Best Hit with DataBase*	*Identity (%)*	*Present in Database?*	*Cross-Contamination?*	*Identity confirmed?*	*Genotype Code*
**BT474 ATCC**	BT474	ok	BT474	100%	yes	no	identity confirmed	AATTAAAAATAAA AATTTTTAWWTTTATAAAAATTTTT TAATTTTWT
**BT474 1986**	BT474	ok	BT474	100%	yes	no	identity confirmed	AATTAAAAATAAA AATTTTTATWTTTATAAAAATTTT TTAATTTTWT

SNP authentication confirmed the identity of both tested BT474 cell line batches and excluded cross-contamination in both.

In order to gain data on short tandem repeat (STR) profiles of both cell lines too, another round of cell line authentication was performed. As depicted in Table 2, also here both cell lines were clearly identified as BT474 cells with all tested loci matching the reference data.

**Table 2:** Cell line authentication reports of STR profiling

*Powerplex16 Loci*	*Database Reference BT474*	*BT474 ATCC*	*BT474 1986*
**AMELO**	X, X	X, X	X, X
**D3**	-,-	17,17	17,17
**THO1**	7,7	7,7	7,7
**D21**	-,-	28,32.2	28, 32.2
**D18**	-,-	13,18	13,18
**PentaE**	-,-	5,5	5,5
**D5**	11,13	11,13	11,13
**D13**	11,11	11,11	11,11
**D7**	9,12	9,12	9,12
**D16**	9,11	9.11	9,11
**CSF**	10,11	10,11	10,11
**PentaD**	-,-	9,14	9,14
**VWA**	15,16	15,16	15,16
**D8**	-,-	10,12	10,12
**TPOX**	8,8	8,8	8,8
**FGA**	-,-	22,25	22,25

All tested gene matched in both BT474 ATCC as well as BT474 1986 cells. The database reference is taken from ATCC; as ATCC uses a 9 gene loci profile only, some loci are left empty.

### Transmitted light microscopy

**Figure 1:**
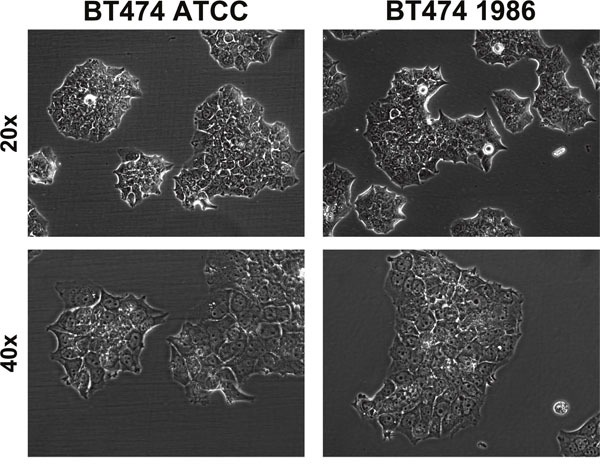
Transmitted light microscopy of BT474 cells Both BT474 cell clones display adherent growth, are “cobblestone-shaped” and grow in clusters that form compact multilayered colonies. Top row: 20x magnification, bottom row: 40x magnification.

Cells were regularly observed under transmitted light microscopy for eventual phenotypic changes. As can be seen in Figure 1, BT474 cells are adherent, “cobblestone-shaped” and grow in clusters that form compact multilayered colonies, rather than growing confluent. Both cell lines displayed similar phenotypes and formed exactly these multilayered colonies.

### Cell growth analysis

**Figure 2:**
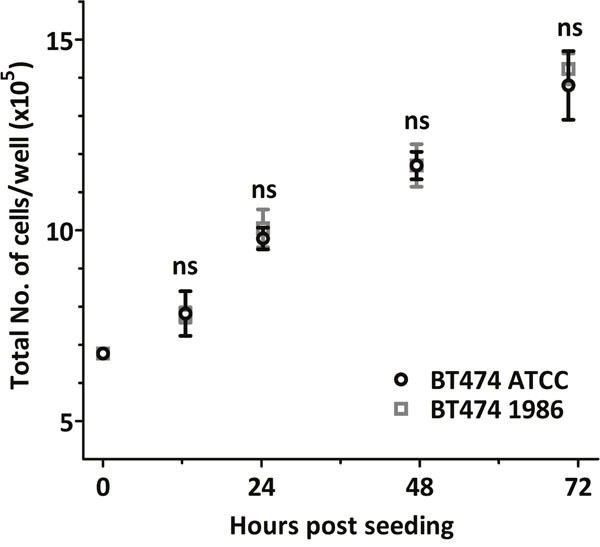
Cell growth analysis Both BT474 cell clones grow at a similar rate, with no statistical differences in cell numbers at 12, 24, 48 and 70 hours after seeding.

In order to detect possible differences in the proliferation rate of the BT474 ATCC and BT474 1986 cell lines, cell growth was analysed. Again, both cell lines behaved similarly, with no statistical differences in cell numbers after 12, 24, 48 or 70 hours (Figure 2).

### Cell surface marker expression and cytokine analysis

**Figure 3:**
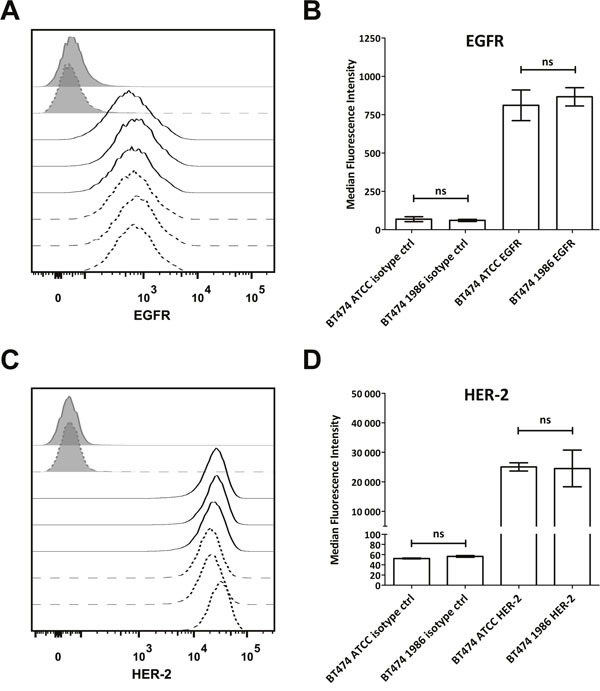
Flow cytometric analysis of EGFR and HER-2 expression **(A)** Both BT474 cell batches display comparable, moderate EGFR expression as detected with cetuximab. Isotype controls are displayed as full grey histograms, cetuximab stainings as lines. Full lines indicate BT474 ATCC cells and dashed lines indicate BT474 1986 cells. **(B)** Statistical readout of flow cytometric experiments shown in **(A)**. Bars depict median fluorescence intensity ± standard deviation. No statistically significant differences could be observed between the two investigated BT474 cell lines with regard to EGFR expression. **(C)** BT474 ATCC and BT474 1986 express high levels of HER-2, detected with trastuzumab. Isotype controls are displayed as full grey histograms, trastuzumab stainings as lines. Full lines indicate BT474 ATCC cells and dashed lines indicate BT474 1986 cells. **(D)** Statistical readout of flow cytometric experiments shown in **(C)**. Bars depict median fluorescence intensity ± standard deviation. Again no statistically significant differences could be observed between the two investigated BT474 cell lines with regard to HER-2 expression.

**Figure 4:**
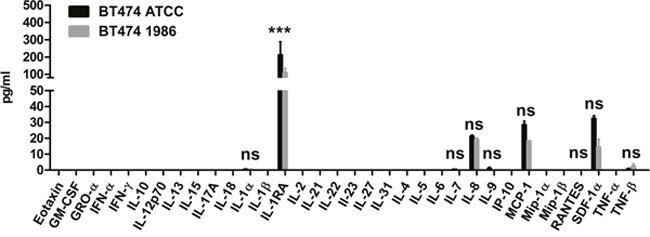
Cytokine, chemokine and growth factor analysis 34 different cytokines, chemokines and growth factors were determined in supernatants of BT474 ATCC and BT474 1986 cells 48 hours in culture. Signals could be detected for IL-1α, IL-1RA, IL-7, IL-8, IL-9, MCP-1, RANTES, SDF-1α and TNF-β, with significantly lower levels of IL-1RA in supernatants of BT474 1986 cells.

In order to further investigate whether also cell surface receptor expression remains stable after such a long storage in liquid nitrogen, human epidermal growth factor receptor-1 (EGFR) and human epidermal growth factor receptor-2 (HER-2) expression was determined, as these cells are most often used for modeling HER-2 or EGFR-driven disease. As can be seen in Figure 3, again both BT474 cells, the ones from ATCC as well as the ones frozen in 1986 showed comparable EGFR and HER-2 expression, indicating no influence of the storage on the expression of these two epidermal growth factor receptors. Furthermore, 34 different cytokines, chemokines and growth factors were analyzed. Here, supernatants of cells 48 hours in culture were investigated via Luminex®. As depicted in Figure 4, only IL-1α, IL-1RA (IL-1 Receptor Antagonist), IL-7, IL-8, IL-9, MCP-1, RANTES, SDF-1α and TNF-β are secreted by BT474 cells. The levels of all other cytokines were under the detection limit of the assay. Both cell lines displayed again similar profiles with the exception of IL-1RA, where BT474 1986 cells had significantly lower levels.

### RNA-profiling

Finally, the whole transcriptome of both cell lines was investigated by means of RNA-profiling. RNA quality and integrity was assessed twice by means of an Agilent 2100 Bioanalyzer (data not shown) prior to analysis in the GeneChip Human Gene 2.0 ST Array. Afterwards several analysis steps were performed to exclude non-biological factors in the variability of the data of the RNA-screens. Firstly, normalization of the input was performed using quantiles method. As can be seen in Supplementary Figure 1, all chips were compared to each other. After normalization, the red line in the graph should be ideally close to zero (indicated as green line). This was well achieved in all tested samples.

Secondly, hybridization data of each chip was graphically analysed in a box plot to gain information about symmetry or possible skewness of the distribution. In all hybridization experiments, the median line was centered between the upper and lower extent of the box and no skewness was detectable (Supplementary Figure 2).

Thus, gene expression data was subsequently analysed for induced and repressed genes. This analysis revealed that only 4 Reference Sequences (RefSeqs) were up-regulated in the BT474 1986 cells compared to the BT474 ATCC cells. These 4 genes are listed in Table 3, ranked by their absolute fold change.

**Table 3:** Up-regulated genes, sorted by their absolute fold change

*Rank*	*Probe Id*	*Gene symbol/Gene name*	*p-value*	*Log(Fold change)*	*Fold change*
3)	16947173	[MME] membrane metallo-endopeptidase;AK291761 AK310664 BC101632BC101658 BC106070 BC143465 BX647134ENST00000360490 ENST00000382989ENST00000460393 ENST00000462745ENST00000462837 ENST00000481828ENST00000491026 ENST00000491597ENST00000492661 ENST00000493237ENST00000493888 ENST00000495577J03779 NM_000902 NM_007287 NM_007288NM_007289 Y00811	1.180e-03	1.521	2.870
12)	16859786	[MIR3188] microRNA 3188; NR_036155	3.417e-02	1.180	2.266
13)	16960698	[MME MME-AS1] MME antisense RNA 1(non-protein coding); ENST00000460393ENST00000484721 J03779 NM_000902NM_007287 NM_007288 NM_007289	8.380e-04	1.135	2.196
19)	17021217	[ME1] malic enzyme 1, NADP(+)-dependent, cytosolic; BC025246 ENST00000369705ENST00000540036 ENST00000541327ENST00000543031 NM_002395	8.336e-06	1.061	2.087

Genes up-regulated in BT474 1986 cells compared to BT474 ATCC cells. Rank indicating position in all differently expressed genes, sorted by absolute fold change.

Accordingly, down-regulated transcripts can be seen in Table 4, again ranked by their absolute fold change. Here, 22 RefSeqs were repressed in the BT474 1986 cells compared to the BT474 ATCC cells.

**Table 4:** Down-regulated genes, sorted by their absolute fold change

*Rank*	*Probe Id*	*Gene symbol/Gene name*	*p-value*	*Log(Fold change)*	*Fold change*
1)	17118180	[CTGF]; BC087839 ENST00000367976NM_001901	1.203e-03	-2.638	-6.224
2)	16703994	[miscellaneous] AF269087	1.636e-02	-1.523	-2.874
4)	17054115	[miscellaneous] ENST00000443280	5.816e-03	-1.488	-2.805
5)	17015324	[NRN1] neuritin 1; BC042019ENST00000244766 ENST00000495850NM_016588	3.099e-03	-1.466	-2.763
6)	17019543	[C6orf223] AK097853; BC032706ENST00000336600 ENST00000439969ENST00000442114 ENST00000448947NM_001171992 NM_153246	2.770e-03	-1.216	-2.324
7)	16660736	[PNRC2] proline-rich nuclear receptor coactivator2; ENST00000471915 NM_017761	2.634e-02	-1.216	-2.323
8)	17120368	[miscellaneous] ENST00000416067	2.502e-02	-1.198	-2.294
9)	16697217	[RNU7-13P RNU7-41P] RNA, U7 small nuclear13 pseudogene; RNA, U7 small nuclear41 pseudogene; ENST00000515917ENST00000516413	1.844e-02	-1.195	-2.289
10)	16793674	[miscellaneous] ENST00000557163;NR_039985	1.521e-02	-1.188	-2.278
11)	17123970	[miscellaneous] ENST00000416067	3.461e-02	-1.185	-2.273
14)	17120362	[miscellaneous] ENST00000416067	2.616e-02	-1.122	-2.176
15)	16961788	[miscellaneous] ENST00000414084;ENST00000432729	1.915e-02	-1.113	-2.163
16)	16956591	[STX19] syntaxin 19; BC034696ENST00000315099 NM_001001850	1.506e-03	-1.068	-2.097
17)	17094355	[miscellaneous] XM_002342917	1.453e-03	-1.066	-2.094
18)	16843998	[miscellaneous] BX647429	3.099e-03	-1.064	-2.090
20)	16989736	[EGR1] early growth response 1;ENST00000239938 ENST00000411801ENST00000535792 M62829 NM_001964	5.112e-03	-1.058	-2.082
21)	16766287	[SNORD59A] small nucleolar RNA, C/D box59A; NR_002737	3.453e-02	-1.054	-2.076
22)	16846081	[miscellaneous] ENST00000575039;NM_001195192	2.877e-02	-1.038	-2.053
23)	16702501	[miscellaneous] ENST00000466451;ENST00000495368	3.861e-02	-1.024	-2.033
24)	16702226	[DKFZp667F0711]; AL713753	4.110e-03	-1.014	-2.019
25)	17123566	[miscellaneous] ENST00000416067	1.891e-03	-1.009	-2.013
26)	16728883	[miscellaneous] ENST00000426191;ENST00000538227	2.507e-02	-1.007	-2.010

Genes down-regulated in BT474 1986 cells compared to BT474 ATCC cells. Rank indicating position in all differently expressed genes, sorted by absolute fold change.

**Figure 5:**
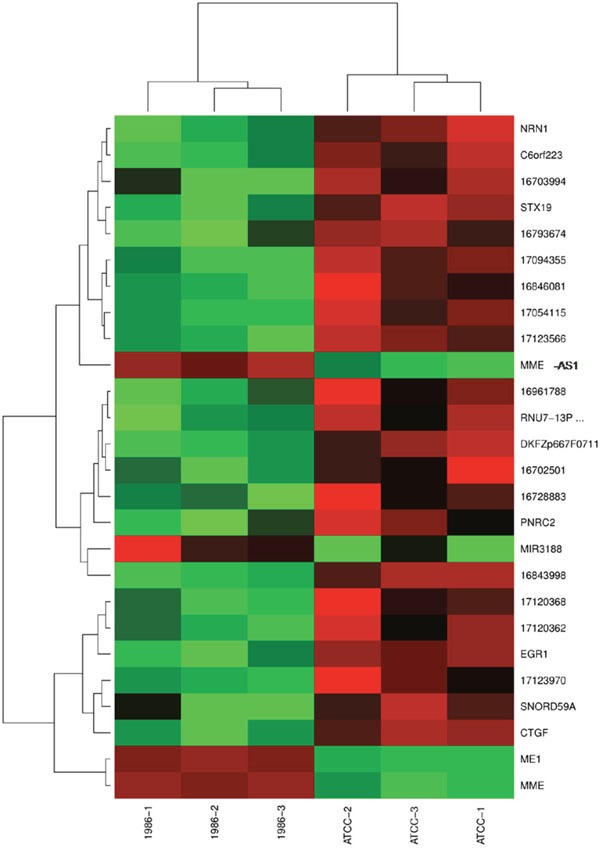
Heat map displaying two-dimensional clustering of the top genes Different samples are labeled on the x-axis. Y-axis displays differently expressed genes. Genes that fall into a cluster showed similar behavior in the experiments. Intensities of expression are represented by red and green, red standing for up-regulation, green for down-regulation and black indicates medium intensities.

For better visualization, differently expressed genes were clustered two-dimensionally in a heat map in Figure 5. Here, genes with similar behavior are compiled in vertical clusters.

Additionally, data was visualized by means of a Volcano Plot displaying the relation between p-values and log2-fold change (Supplementary Figure 3). The complete list of all signals from the RNA profiling can be found in the supplementary data (Supplementary Table 1).

## DISCUSSION

BT474 cells are routinely used in our laboratory as a model system for HER-2 overexpressing breast cancer. These cells were isolated in 1978 by Lasfargues et al. from a patient with solid, invasive ductal breast carcinoma [16]. Prior to the introduction of this cell line, most human breast cancer cell lines were derived from pleural effusions, which accented BT474 cells as a more valid surrogate for solid tumors. Later, when breast cancer research focused on HER-2, BT474 cells, which highly over-express this receptor, became very important as mimic for HER-2 driven disease [17–18]. Moreover, BT474 cells grow well in immunocompromised mice, making this cell line also valuable for *in vivo* xenograft studies [19–21].

If a cell line is constantly used in a lab since 1978, only two variants of currently used clones can exist: a) clones that have been in culture for numerous passages, either constantly, or (more likely) by means of repeated freezing and thawing or b) clones that have been frozen originally as master stock and were stored for long periods of time. For the first variant, a great deal of investigation was performed to evaluate the effects of repeated cell divisions as well as effects of freezing and thawing on cell lines. On the latter condition only little research has been performed, most likely because it is not so easy to access cell clones that have been in the same liquid nitrogen container for more than 20 years.

Fortunately, we could get access to a BT474 clone, which has been stored for 28 years under optimal conditions, not harmed by unforeseeable events such as e.g. electrical breakdowns, relocation of laboratory space or being discarded when research topics change.

Our internal cell line quality control standards comprise of regular authentication via SNP profiling. In this particular case with such a long history of storage, however, we did not want to solely rely on one authentication run. That is why we analyzed this cell clone also by STR profiling and thoroughly monitored growth behavior, eventual phenotypic changes, as well as cell surface receptor expression. As these parameters were stable (Table 1, 2; Figures 1, 2 & 3), we decided to further investigate possible changes in gene expression via a human RNA profile array.

Interestingly, only 4 reference sequences were significantly induced in the ‘1986’ clone (see Table 3), among these MME (membrane metallo-endopeptidase, or neprilysin and CD10, as it is alternatively termed), which is linked to higher tumor grade and poor prognosis upon expression in invasive breast carcinoma [22]. Interestingly, also the MME-antisense RNA 1 was induced, too.

Another up-regulated gene encodes the NADP(+) dependent cytosolic malic enzyme, which is physiologically found in liver and white adipose tissue [23], but was also described in human breast, colon, lung and lymphoid cancer cell lines, where it is involved in the conversion of excess mitochondrial malate [24]. The last induced RNA sequence was the microRNA3188, which has been described in melanoma [25].

By contrast, 22 reference sequences were repressed; the gene, which showed the highest fold change overall was CTGF (connective tissue growth factor), which was more than 6-fold down-regulated. CTGF can induce tumor cell epithelial-mesenchymal transition (EMT) in breast cancer, and is able to promote tumor growth and metastasis, resulting in poor clinical outcomes [26]. The second most down-regulated gene was neuritin 1 (NRN1), which has been proposed as hypoxic marker and potential marker for tumor angiogenesis [27], followed by the proline-rich nuclear receptor coactivator 2 (PNRC2), syntaxin 19, and early growth response 1 (EGR1). Syntaxin 19 (or epimorphin, as it is also termed) was at least in the murine system associated with the promotion of alveolar hyperplasia and mammary adenocarcinoma [28]. Also EGR1 was described to be involved in breast tumorigenesis via transcriptional control of miR-20b, which can function as an oncogene via tumor suppressor targeting [29].

Additonally, a small nuclear pseudogene, RNU7-13P and a small nucleolar RNA were repressed. The last 15 down-regulated RNAs were the Chromosome 6 Open Reading Frame 223, miscellaneous RefSeqs or coded for an uncharacterized gene (DKFZp667F0711).

Finally all 26 modulated RefSeqs were clustered in a heat map (Figure 5). Although some clusters can be detected, no striking difference between the two cell line conditions can be observed.

Considering that 40,716 RefSeq transcripts were analyzed and only 26 were differently expressed, one can conclude that even this decade-long storage resulted in only minor changes to the original tumor cell line of ATCC. However, one has to take into consideration that also the ATCC clone has had to be stored for long periods of time, albeit in much better controlled conditions than in common laboratories. Thus, it is unfortunately only practically possible to compare cells to batches of ATCC's carefully controlled ‘seed stock’, cells descending early from the original cells, but never to the cells at the time point of establishment. Cell repositories such as ATCC perform a systematic seed-stock cell-banking method, with low-passage, contaminant free and repetitively authenticated stocks in order to produce virtually identical distribution lots.

As it is not feasible to buy new cell lots for every new experiment, long term storage is a necessary evil; in this study we could demonstrate at least for BT474 cells that it is a compromise that can be made, as it affects tumor cell lines only moderately. However, also such minor changes should be carefully assessed before the start or continuation of experiments, especially if also phenotypic alterations can be observed.

## MATERIALS AND METHODS

### Cell lines and monoclonal antibodies

BT474 cells were either obtained from ATCC (American Type Culture Collection - ATCC, Rockville, Maryland, USA; Cat. No: HTB-20^®^) or were stored in a liquid nitrogen tank in the lab of Prof. Dr. Thomas Grunt from the Institute for Cancer Research of the Medical University of Vienna. Prof. Grunt's cells were cryo-conserved in 1986, which is why all experiments with these particular cells described in this paper are labeled as ‘BT474 1986’. Both BT474 cell lines were kept in α-MEM medium supplemented with 10% fetal calf serum (FCS), 2 mM l-glutamine, penicillin (100 U/mL) and streptomycin (100 μg/mL) and were grown in a humidified atmosphere of 5% CO_2_ at 37°C.

The anti-HER-2 monoclonal antibody trastuzumab (Herceptin®) was purchased from Roche (Hertfordshire, United Kingdom) and the anti CD20 monoclonal antibody rituximab (MabThera®, Rituxan®) likewise. Cetuximab, an anti-human EGFR antibody was purchased from Merck KGaA (Darmstadt, Germany).

As detection antibody goat anti-human IgG AlexaFluor488 (AF488, Invitrogen, Life Technologies, Grand Island, New York, USA) was employed.

### Cell line authentication

Cell lines were sent for Authentication prior to all experiments to Multiplexion GmbH (Heidelberg, Germany). Cell identity was confirmed by SNP-profiling. In order to test the cells with both established cell line authentication methods, DNA was additionally sent for STR-profiling to LGC Standards (LGC Standards GmbH, Wesel, Germany) and investigated via their Power Plex® 16HS 16 Loci service.

### Transmitted light microscopy

Cell culture flasks were observed under transmission light microscope (Zeiss Axio Observer Z1, Carl Zeiss AG, Jena, Germany). Pictures were acquired between cell passages 3 to 5 at 20x and 40x magnification.

### Cell growth analysis

Both BT474 cell lines were seeded in 6-well plates in triplicates at a density of 0.75×10^5^ cell/cm^2^, totaling 6.77×10^5^ cells/well. After 12, 24, 48 and 70 hours, cells were harvested and measured using the TC10™ Automated Cell Counter (Bio-Rad Laboratories, Hercules, California, USA).

### Flow cytometry

For the flow cytometric assessment of EGFR and HER-2 expression, single-cell suspensions of both BT474 cell lines were incubated with 200μl of either 10μg/ml cetuximab, trastuzumab or rituximab for 30 min at 4°C, followed by two washes in ice cold flow cytometry buffer (FACS-buffer, phosphate buffered saline (PBS) + 5% normal goat serum) and incubation with anti-human IgG AlexaFluor488 (AF488) antibodies (200μl of 10μg/ml, Invitrogen, Life Technologies, Grand Island, New York, USA) for 30 min at 4°C. After washing twice with ice cold FACS-buffer, samples were analyzed on a 3-laser FACSCanto™ II (BD Biosciences, Franklin Lakes, New Jersey, USA). 20,000 single cells were recorded and data was subsequently analyzed using the FlowJo V10.4 software (Tree Star, Inc., Ashland, Oregon, USA).

### Cytokine, chemokine and growth factor profiling

In order to investigate the cytokine, chemokine and growth factor profile of both BT474 cell lines, cells were seeded in 6-well plates at a density of 0.75×10^5^ cell/cm^2^. Supernatants were harvested after 48 hours and analyzed with the ProcartaPlex™ Human Cytokine & Chemokine Panel 1A (34 plex) immunoassay (Affymetrix, eBioscience, Thermo Fisher Scientific Inc., Vienna, Austria) according to the manufacturer's instructions. This ProcartaPlex assay uses the Luminex® xMAP technology for the multi-analyte detection of the following 34 cytokines, chemokines and growth factors: Eotaxin, GM-CSF, GRO-α (CXCL1), IFN-α, IFN-γ, IL-10, IL-12p70, IL-13, IL-15, IL-17A, IL-18, IL-1α, IL-1β, IL-1RA (IL-1 Receptor Antagonist), IL-2, IL-21, IL-22, IL-23, IL-27, IL-31, IL-4, IL-5, IL-6, IL-7, IL-8, IL-9, IP-10 (CXCL10), MCP-1 (CCL-2), Mip-1α, Mip-1β, RANTES (CCR5), SDF-1α (CXCL12α), TNF-α and TNF-β.

### RNA-profiling

RNA of 3 independent biological replicates of each BT474 cell line (‘ATCC’ and ‘1986’, respectively) was isolated. Thus, cells between passage 5 and 8 were lysed and homogenized in Trizol® (Life Technologies (Invitrogen), Vienna, Austria), followed by a centrifugation step to isolate the aqueous phase containing total RNA. Subsequently, the RNA-containing solution was loaded onto RNeasy Mini spin columns (Qiagen, Hilden, Germany) and processed according to the manufacturer's instructions. An additional on-column DNase-treatment was performed (Qiagen, Hilden, Germany). RNA yields were measured with a NanoDrop ND-1000 (Peq-lab, Erlangen, Germany). RNA quality and integrity was assessed by means of an Agilent 2100 Bioanalyzer (Agilent, Santa Clara, California, United States of America). Gene expression profiling was carried out at Atlas Biolabs (Berlin, Germany), where RNA quality was again assessed by means of Agilent 2100 Bioanalyzer (Agilent, Santa Clara, California, United States of America). Having proved good RNA quality of all samples (RIN value > 9.5; RIN = RNA Integrity Number, 1 stands for completely degraded RNA, 10 for intact RNA [30]), Expression Profiling was performed, using the GeneChip Human Gene 2.0 ST Array (Affymetrix, Santa Clara, California, United States of America), which tests 40,716 RefSeq transcripts. Sample analysis was also conducted at Atlas Biolabs. Here, quality control steps of chip data comprised of normalization and transformation in order to exclude non-biological factors (e.g. amount of stain applied) which could contribute to variability of data. Normalization was performed in order to reduce unwanted variation across chips. Thus, the input data was normalized by means of quantiles method.

After these quality control steps, gene expression data was statistically analyzed.

### Data handling and statistics

Cell growth rate measurements were performed in biological triplicates. Two-way ANOVA in conjunction with Bonferroni-post test correction was performed in order to detect statistical differences between the cell growth of the two cell lines.

Flow cytometric measurements were done in 3 biological replicates and tested for statistical differences via one-way ANOVA along with Tukey's multiple comparison test.

Cytokine expression experiments were also performed in 3 biological replicates. Data was again analyzed by means of two-way ANOVA in conjunction with Bonferroni-post test correction.

The output data of the Human Gene Arrays was computed as fold change and as log-fold change. Afterwards genes were sorted by absolute fold change. Data was analyzed by means of t-Test.

Statistic calculations were performed using GraphPad Prism 5 Software (GraphPad Software, Inc., La Jolla, California, United States of America) and statistical significance was accepted at p<0.05 (*), p<0.01 (**) and p<0.001 (***).

## SUPPLEMENTARY MATERIALS FIGURES AND TABLES





## Abbreviations

ATCC: American Type Culture Collection
EGFR: Epidermal Growth Factor Receptor
GM-CSF: Granulocyte Macrophage Colony-Stimulating Factor
HER-2: Human Epidermal Growth Factor Receptor-2
IL: Interleukin
TNF: Tumor Necrosis Factor
RefSeq: Reference Sequence
SNP: single nucleotide polymorphism
STR: short tandem repeat profiling
