# Supplementary material for: Long term storage in liquid nitrogen leads to only minor phenotypic and gene expression changes in the mammary carcinoma model cell line BT474
Source: Oncotarget. 2017 Mar 28;8(21):35076–87. doi: 10.18632/oncotarget.16623 (PMC5471036; doi:10.18632/oncotarget.16623)
Supplement: Supplementary file 1 [file oncotarget-08-35076-s001.pdf]

Long term storage in liquid nitrogen leads to only minor phenotypic and gene expression changes in the mammary carcinoma model cell line BT474

SUPPLEMENTARY MATERIALS

SUPPLEMENTARY FIGURES AND TABLE

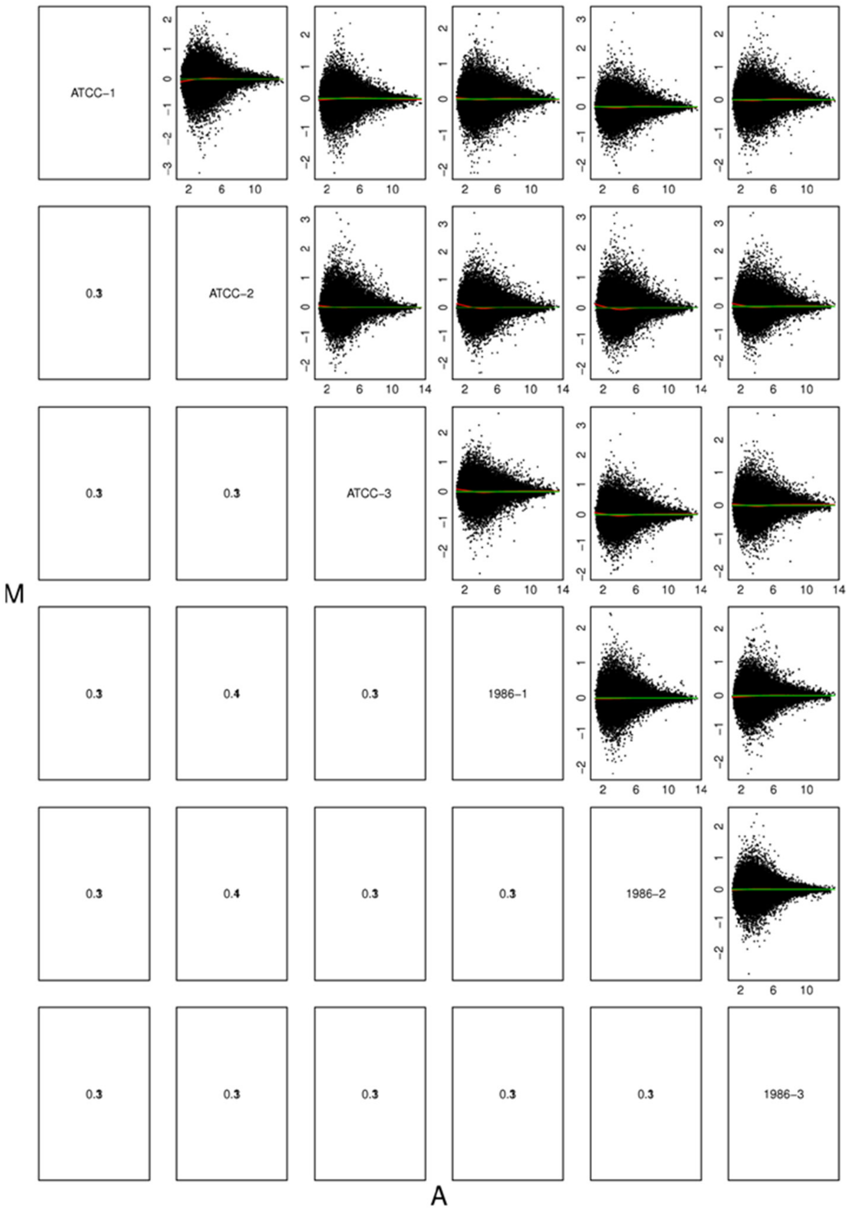

Supplementary Figure 1: Chip-to-Chip comparison after normalization. Plots on the diagonal indicate the names of the compared chips. Plots above the diagonal display the comparison of data of the two chips. Ideally after normalization the red line should be very close to zero, indicated as green line. Plots below the diagonal indicate the variance of the ratios between the two chips compared. If the compared chips are identical, the variance equals zero.

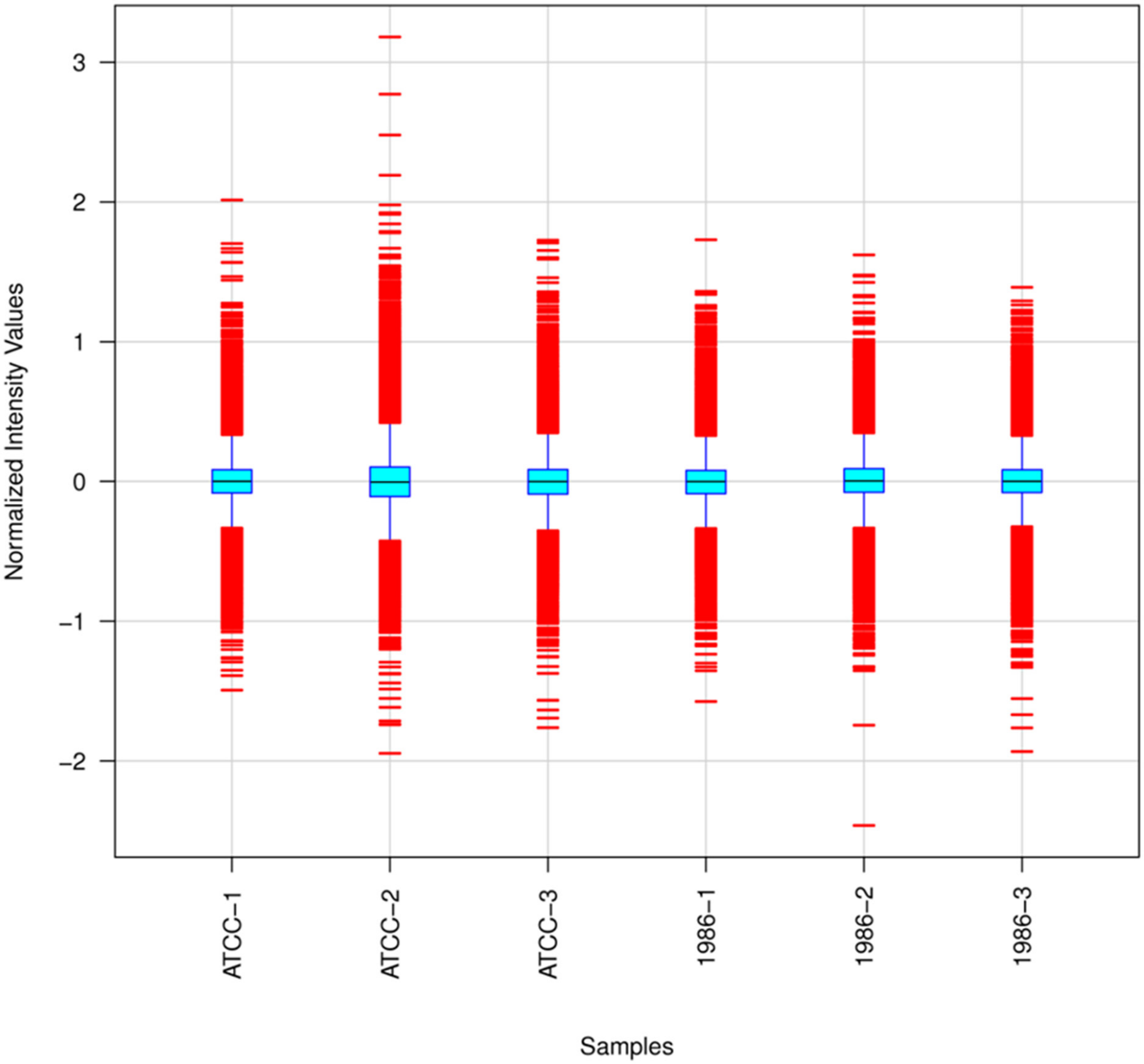

Supplementary Figure 2: Box plots of hybridization data. Boxes represent the 25th and 75th percentile, respectively; the line in the middle represents the median. Whiskers end in the minimum and maximum values (no more than 1.5. fold of box-lengthes remote from the edge of the box). Data of every chip are centered and no skewnesses are detectable.

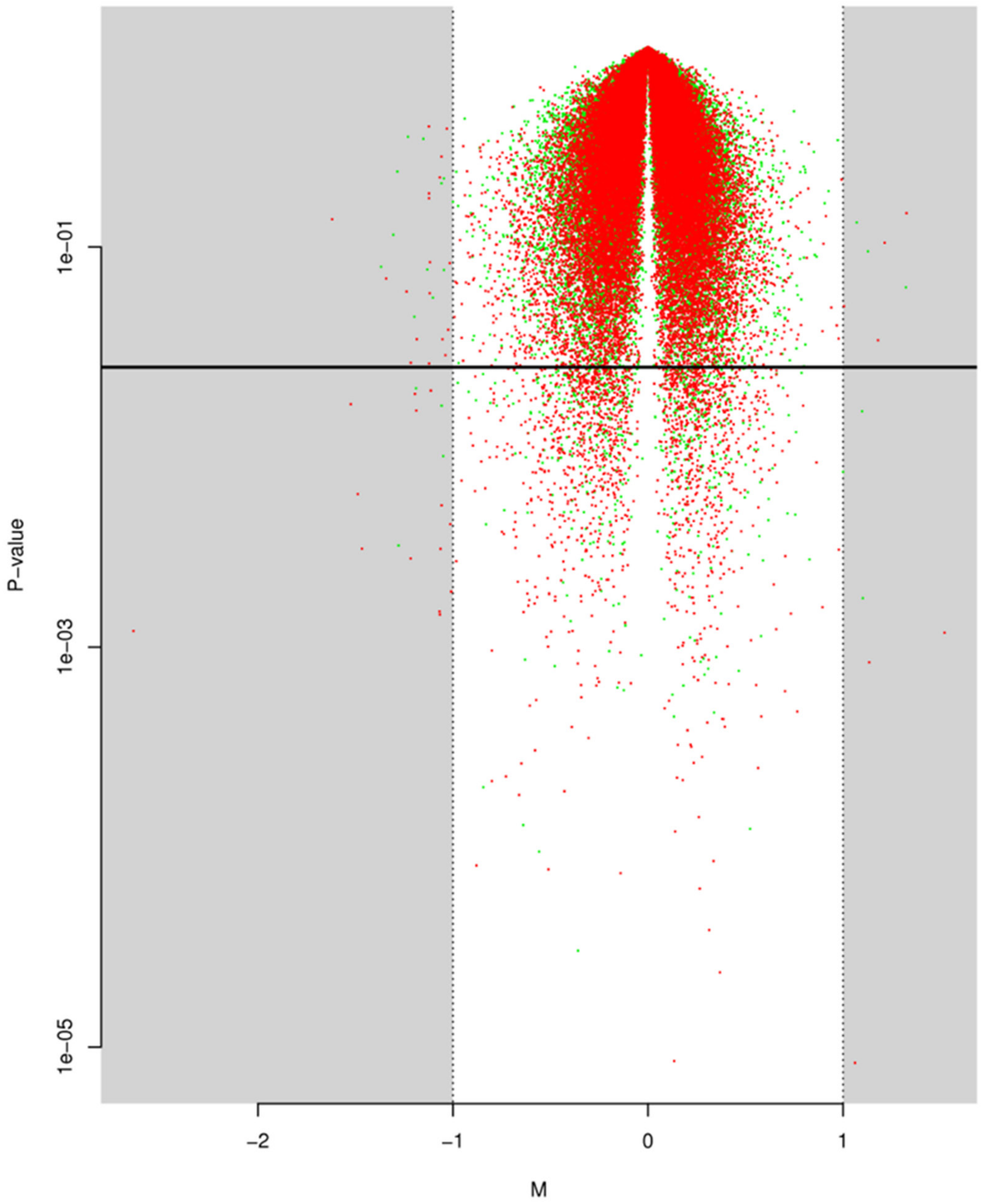

Supplementary Figure 3: Volcano Plot displaying the relation between *p-values* and *log*$_2$-fold change. P-Values are displayed on the y-axis on a log scale; *p-value* cutoff is at 5%. Original data is displayed in red, randomly categorized data in green.

Supplementary Table 1: Data set of all signals from RNA-profiling of BT474 ATCC and BT474 1986 cells.
This Excel sheet displays all signals obtained from RNA-profiling of BT474 ATCC and BT474 1986 cells

See Supplementary File 1
